# The Bottleneck of Central Processing: Clues from Reaction Times

**DOI:** 10.1371/journal.pbio.0030084

**Published:** 2005-02-08

Between stimulus and response lies a black box—the mind—whose inner workings are largely unmapped. One of the essential questions about those inner workings concerns the serial versus parallel nature of their processing capabilities. Parallel processing allows multiple tasks to proceed at once, while serial processing creates a bottleneck through which multiple tasks must pass, one at a time. Any reasonably complex task is likely to involve both parallel and serial components, and parsing a task into its components is a central goal for researchers of cognitive processing. In a new study, Mariano Sigman and Stanislas Dehaene propose a model of cognitive processing for a set of simple tasks in which a bottleneck occurs between initial sensory processing and motor response. They predict that this bottleneck will contribute significantly to variations in response time as the cognitive challenge increases and verify this by testing people on a specific numerical evaluation task.

A simple but highly effective technique for examining bottlenecks is to measure variations in response time for a task as the stimulus is varied in some small but cognitively challenging way, or when the stimulus is presented along with a stimulus for a competing task. The task in this study was to determine whether a presented number was greater than or less than 45. The complexity of the task was determined by three variables: notation, distance, and response complexity. Notation was varied by presenting the number either as a numeral or its spelled-out equivalent (for instance, “36” or “thirty-six”). Distance was varied by presenting numbers either closer to or further from 45 (for instance, 31 versus 36), and the required response was either one or two finger taps. In a separate series of experiments, the researchers challenged the subject with an interfering tone-recognition task at the same time as or slightly after the presentation of the numerical task.[Fig pbio-0030084-g001]

**Figure pbio-0030084-g001:**
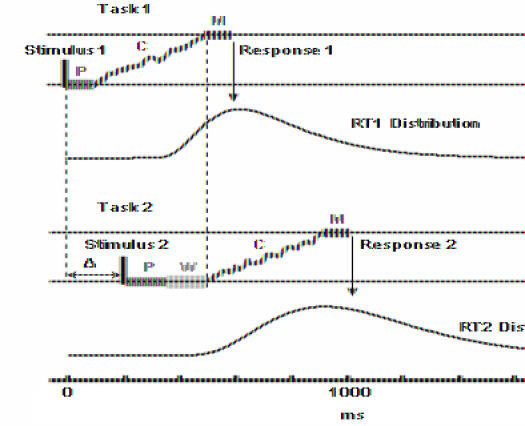
Comparing human reaction times

The use of these two sets of experiments allowed the authors to deduce two different kinds of information about the processing involved in the number task. First, by varying the delay in presentation of the tone-interference task and measuring the delay in response time for the number task, they showed that both number perception and motor response can proceed in parallel with other, competing tasks, while the central component of the number task—determining distance—was processed serially, through a central bottleneck.

Next, Sigman and Dehaene turned off the tone, and asked how variations in notation, distance, and response complexity altered the variance in the response time—that is, the spread of values for the same task by the same subject. For all three variables, the more challenging task (numbers presented as words, smaller distance, or two taps) had an increased response time. However, only the calculation of distance increased the spread of values obtained in multiple trials. This further suggests that only the central calculation step—what the authors refer to as stochastic integration—proceeds through a central bottleneck, while the other two components can be processed in parallel. Thus, in both experiments, the task was parsed by the brain into the same components, with the serial component being the one subject to the most variance.

Sigman and Dehaene note that the ability of the perceptual and motor parts to be performed without central computation depends on the extreme simplicity of the tasks in this experiment. More complex motor challenges, for instance, undoubtedly would require some central input, and thus proceed through the bottleneck. Similarly, a high degree of training in the numerical distance task would likely increase the automaticity of the response, thus avoiding the central slowing seen in the task-naïve subjects studied here.

